# *Pseudomonas luteola* Bacteremia in Newly Diagnosed Systemic Lupus Erythematosus

**DOI:** 10.1155/2021/4051378

**Published:** 2021-08-14

**Authors:** Mazin Barry

**Affiliations:** Division of Infectious Diseases, College of Medicine, King Saud University, Riyadh, Saudi Arabia

## Abstract

*Pseudomonas luteola* is rarely considered as a human pathogen.There are only fewer than twenty reported cases of *P. luteola* infections since 1950. It has been described in both immunocompromised and immunocompetent patients as a cause of both nosocomial and community-acquired infections. We report a rare case of *P. luteola* infection in a previously healthy patient who was admitted to hospital with a first presentation of Systemic Lupus Erythematosus (SLE) presenting with *P. luteola* bacteremia.

## 1. Introduction

*Pseudomonas luteola* is a gram-negative bacillus that rarely causes human disease, although it has been reported as a cause of pancreatitis, endocarditis, pyocele, and brain abscesses in few isolated case reports [1–6]. To date, *P. luteola* was only reported to cause infection in a patient with systemic lupus erythematosus (SLE) in 1982 in Tel Aviv, Israel [3]. We report the second case of *P. luteola* bacteremia in a patient newly diagnosed with SLE.

## 2. Case Report

A 19-year-old male patient who was not known to have any medical illness before presented to our institution's emergency room complaining of fever for one-week, with five days of left flank pain and burning micturition. He also reported a history of recurrent joint pain with swelling and redness over the last two months prior to his presentation, with a history of few self-limited episodes of productive cough with mild hemoptysis over the last month associated with pleuritic chest pain. He did not report any skin rash, or any neurological symptoms, and he had no photosensitivity or reported oral ulcers. He lives with his parents in a rural village in Saudi Arabia, in a low-socioeconomic neighborhood with poor sanitation. He reported several occasions of walking barefoot on sewage-contaminated grounds.

Physical examination revealed a sick-looking, pale, and exhausted young man.His blood pressure was 111/59 mmHg, pulse 115 beats/min, temperature 39.4°C, and respiratory rate 20 breaths/min. His eyes were red with minimal discharge; he had a few healed oral ulcers and bilateral submandibular lymph nodes measuring less than 2 cm. The abdomen showed hepatosplenomegaly, and both the liver and spleen were palpated at 2 cm below the costal margins; he also had left renal angle tenderness. Chest and cardiovascular examination showed a grade 2/6 midsystolic ejection murmur on the right upper sternal border, and his lung fields were clear with no rub.He had no skin rash, nor any joint swellings.

Laboratory results were as follows: WBC 4.1 × 10^9^/L, RBC 4.29 × 10^12^/L, Hb 124 g/L, and Plt 140 × 10^3^/L. The ESR was 38 mm/hr. Serum creatinine was 158 umol/L, urea 12.8 mmol/L, with normal coagulation profile and liver function test (LFT): AlP 78 u/L, AST 41 u/L, ALT 60 u/L, and GGT 58 U/L. Urine analysis was significant for WBC 80, RBC 40, blood +2, and haemoglobin +2 with few granular casts, but no protein, glucose, ketone, nor nitrite, and his chest X-ray was normal. Blood and urine cultures were collected upon first presentation.

He was resuscitated in the emergency room and then admitted to hospital with a provisional diagnosis of a urinary tract infection. He was started on cefuroxime 750 mg IV every 8 hrs.

On day two of admission, he had several spikes of fever between 38.5 and 39.2°C; his WBC dropped to 3.1 × 10^9^/L with a neutrophil count of 1.44 × 10^9^/L, RBC 4.00 × 10^9^/L, Hb 114 g/L, and Plt 88 × 10^3^/L. His renal profile improved: creatinine 90 umol/L, urea 7.4 mmol/L, and repeated urine analysis showed microscopic hematuria with 111 RBCs/mm^3^. Sputum gram and acid-fast stains showed no pathogens nor acid-fast bacilli, respectively. Transesophageal echocardiography was normal with no valvular abnormality. Abdominal CT confirmed hepatosplenomegaly and excluded renal stones, obstruction, and hydronephrosis.

On day three of admission, he had three spikes of fever, 38.5, 38.8, and 39.0°C. His WBC continued to drop to 2.9 × 10^9^/L with a neutrophil count of 1.42 × 10^9^/L, RBC 4.04 × 10^9^/L, Hb 116 g/L, and Plt 91 × 10^3^/L. His renal profile showed creatinine 95 umol/L, urea 4.8 mmol/L. Urine culture did not grow any organisms.

On day four of his hospital stay, he had two spikes of fever, 38.2 and 38.8°C.His WBC continued to drop to 2.0 × 10^9^/L differential showing neutrophil count 1.26 × 10^9^/L, RBC 3.71 × 10^12^/L, Hb108 g/L, and Plt 80 × 10^3^/L. Creatinine was 99 umol/L, urea 4.1 mmol/L. Repeated urinalysis was still showing microscopic hematuria of 390 RBCs/mm^3^ and protein +1 with 10 WBC casts. Serologies for CMV, EBV, HIV, HBV, HCV, *Brucella* sp., and *S. typhi* were all negative, and thick and thin films for malaria were negative. His ANA was 1 : 640, his anti-Ds DNA was 1035 i.u/ml, and his C3 0.24 g/L and C4 0.0312 g/L, normal range: 0.9–1.8 g/L for C3 and 0.1–0.4 g/L for C4. Two of the blood cultures sent on the initial day of hospitalization were flagged by the BacT/ALERT^®^ 3D system, and direct gram stain showed gram-negative bacilli; after being subcultured the isolate showed growth in blood and MacConkey agars. Gram stain from the plates also showed gram-negative bacilli, biochemical tests revealed that the organism was oxidase negative.Inspecting the colonies on MacConkey agar showed that it was a nonlactose fermenter; further identification and susceptibility testing was performed by using microscan “Walk AWAY 96 Plus” that confirmed the isolate to be *Pseudomonas luteola*. Cefuroxime was changed to ceftriaxone 2 gm IV OD.

**Figure 1:**
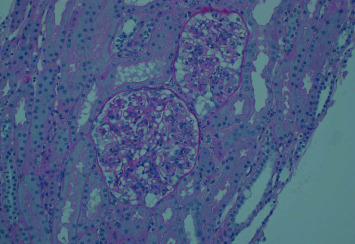
Light micrograph showing the glomerulus with global involvement of endocapillary and mesangial hypercellularity and matrix expansion, influx of leukocytes, and occasional double contours consistent with lupus nephritis class IV-G (hematoxylin and eosin stain).

On day five of hospital admission, the patient continued to have three spikes of fever, 37.9, 38.5, and 39.0°C, with chills and rigors. His WBC dropped to its lowest level since admission to 1.5 × 10^9^/L with a differential showing neutrophil count of 0.47 × 10^9^/L, RBC 3.77 × 10^9^/L, Hb 107 g/L, and Plt 65 × 10^3^/L. Creatinine was 94 umol/L, urea 3.7 mmol/L. Bone marrow aspiration and biopsy demonstrated a reactive bone marrow suggesting that his pancytopenia was due to peripheral destruction. Results of the bacterial susceptibility showed that the isolate was susceptible to amikacin, amoxicillin, aztreonam, cefepime, ceftazidime, cefotaxime, ceftriaxone, ciprofloxacin, gentamicin, imipenem, piperacillin/tazobactam, and tobramycin but resistant to cefuroxime. He was continued on ceftriaxone and gentamicin was added. G-CSF was started because of the pancytopenia. Renal biopsy revealed diffuse proliferative glomerulonephritis (Figure 1) with 15% fibrosis and 9/24 activity index. Based on the history of arthritis, the positive ANA, positive anti-ds-DNA, recurrent chest pain (serositis), pancytopenia, and nephritis, he was diagnosed with Systemic Lupus Erythematosus (SLE). Methylprednisolone intravenously was subsequently started. From day 6 to 10 of admission, he was continued on ceftriaxone, gentamicin, and methylprednisolone, he showed remarkable improvement clinically, all his symptoms resolved, and his fever subsided. Repeated blood cultures taken on day five of admission showed no growth, and his CBC improved, WBC reached 8.3 × 10^9^/L, RBC 3.86 × 10^12^/L, and Plt 91 × 10^3^/L. Gentamicin was discontinued. He completed 14 days of ceftriaxone IV and was discharged on ciprofloxacin 500 mg PO every 12 hours for 7 days and prednisolone 60 mg PO with instructions for tapering. Three months later, he was seen in clinic, he was doing well, with no symptoms, normal CBC and creatinine, and no RBC in his urine.He was maintained on mycophenolate mofetil, prednisolone, and lisinopril. At two-year follow-up, nephritis was stable on that management, his WBC count remained normal, and he never suffered from any recurrence of any bacteremia and never had major flare-up of his SLE.

## 3. Discussion

*Pseudomonas luteola* is an uncommon opportunistic pathogen. It was previously known as CDC group Ve-1 and *Chryseomonas luteola*. *P. luteola* infections are often associated with foreign bodies such as central venous and peritoneal dialysis catheters. Reported infections include bacteremia, peritonitis (associated with appendicitis and colon cancer, as well as catheters), osteomyelitis, endocarditis, leg ulcers, cellulitis, postoperative endophthalmitis, and meningitis.

Microbiologically, *Pseudomonas luteola* are aerobic, non-spore-forming, gram-negative bacilli, and they are motile due to the presence of one or more polar flagella. They are lactose nonfermenters and grow well on MacConkey agar. Most clinical isolates are oxidase negative. It produces yellow-pigmented colonies on MacConkey agar that help distinguish them from other pseudomonas. Unlike other fluorescent pseudomonads including *P. aeruginosa, P. fluorescens, and P. putida,* they do not reduce nitrate and oxidize xylose [1]. It is believed to be a saprophyte mainly inhabiting soil and water, but it has been reported in few case reports as a cause of some human infections including bacteremia, pneumonia, biliary tract infections, surgical site infections, abscesses, peritonitis, subdural empyema, and infections associated with the presence of prosthetic devices (Table 1) [4]. Although almost all previous reported patients with *P. luteola* infections were immunocompromised, had prosthesis, or indwelling catheters, there are three cases reported in previously healthy patients. Our case is the forth case of *P. luteola* infection ever described in a healthy patient [9, 12]. Engel et al. [8] described a previously healthy patient who presented with fever and raised liver enzymes. Rastogi and Sperber described a 60-year-old previously healthy patient who presented with *P. luteola* bacteremia and cellulitis and had initially high LFTs but reverted to normal prior to hospital discharge [9]. Dalamaga et al. in 2004 described a *P. luteola* gluteal abscess and bacteremia that followed two prior intramuscular injections of acetaminophen [12].

Clinical isolates of *P. luteola* are often resistant to first- and second-generation cephalosporins, tetracyclines, ampicillin, and trimethoprim-sulfamethoxazole but are susceptible to third-generation cephalosporins, mezlocillin, imipenem, aminoglycosides, and quinolones.Our isolate was similarly resistant to cefuroxime and susceptible to third-generation cephalosporins. The fact that he was previously healthy and cultures grew the organism from his blood that was taken on the day of admission all suggest that the source of the bacteria was the community, in contrast to the nosocomial source reported in most previous studies [2].

Among all cases reported, only one was a *P. luteola* septicemia in a known systemic lupus erythematosus patient [3].The patient was a 50-year-old immunocompromised female on steroids for years and had an indwelling drain for over 6 weeks for hemorrhagic pancreatitis and pancreatic abscess; our case, on the contrary, was not known to have SLE before the current admission and was on no medications. We hypothesize that the patient acquired *P. luteola* from the poor sanitary environment in the area where he lived, and the initial presentation of SLE weakened his immunity making him susceptible to *P. luteola* primary bacteremia. To the best of our knowledge, our case is the first to be reported in a nonimmunocompromised patient with primary bacteremia with no other focus of infection. It is also worth mentioning that this is the first case of *P. luteola* infection in any Gulf Cooperation Council (GCC) countryto be reported thus far. This case, in conjunction with previous reports, indicates that this saprophytic organism might be emerging as a human pathogen. It also advocates educating all lab personnel to avoid discarding *P. luteola* as a contaminant as it can be a cause of both nosocomial and community-acquired infections. Finally,it is difficult to determine if this type of bacteremia in newly diagnosed SLE patients may be the trigger for first presenataion and subsequent SLE flares, suggesting further studies on bacteremia in SLE to investigate this phenomenon.

## Figures and Tables

**Table 1:** *Pseudomonas luteola* infections reported in the literature with the source of the bacteria and the disease caused/associated with it.

Infection	Microbiological source	City/country	Year reported	Reference
Endocarditis	Blood, femoral artery thrombus	Marseille, France	2005	Casalta et al. [7]
Endocarditis	Blood	Rabat, Morocco	2004	Chihab et al. [5]
Pancreatitis	Blood	Tel Aviv, Israel	1983	Berger et al. [3]
Septicemia	Blood	Rabat, Morocco	2010	Ngoh et al. [2]
Neonatal sepsis	Blood	Rabat, Morocco	2004	Chihab et al. [5]
Bacteremia	Blood	Hines, United States of America	1987	Engel et al. [8]
Pyocele	Blood, tissue	Hyderabad, India	2010	Ramana et al. [4]
Multiple brain abscesses	Stereotactic aspiration	France	2009	Gaschet et al. [6]
Facial cellulitis	Blood	New Jersey, United States of America	1998	Rastogi and Sperber [9]
Femur abscess	Tissue	Israel	1995	Rahav et al. [10]
Leg ulcer	Blood and skin biopsy	Greece	2002	Tsakris et al. [11]
Cutaneous abscess	Blood and skin biopsy	Athens, Greece	2004	Dalamaga et al. [12]
Endophthalmitis	Vitreous humor fluid	Makati City, Philippines	2009	Uy et al. [13]
Biliary infection	Bile	Mumbai, India	2010	De et al. [1]
Catheter-related bloodstream infection (1)	Blood	Lyon, France	2013	Otto et al. [14]

## Data Availability

All data are available upon reasonable request from the corresponding author.
